# Respiratory syncytial virus in young children: community cohort study integrating serological surveys, questionnaire and electronic health records, Born in Bradford cohort, England, 2008 to 2013

**DOI:** 10.2807/1560-7917.ES.2021.26.6.2000023

**Published:** 2021-02-11

**Authors:** Ania Zylbersztejn, Lucy Pembrey, Harvey Goldstein, Guy Berbers, Rutger Schepp, Fiona van der Klis, Charles Sande, Dan Mason, John Wright, Rosalind Smyth, Pia Hardelid

**Affiliations:** 1 Population, Policy & Practice Research and Teaching Department, UCL Great Ormond Street Institute of Child Health, London, United Kingdom; 2 Medical Statistics Department, London School of Hygiene and Tropical Medicine, London, United Kingdom; 3 Centre of Infectious Disease Control, National Institute of Public Health and the Environment (RIVM), Bilthoven, the Netherlands; 4 Kemri-Wellcome Trust Research Programme, Kilifi, Kenya; 5 Bradford Institute for Health Research, Bradford Teaching Hospitals NHS Foundation Trust, Bradford, United Kingdom; 6 Infection, Immunity and Inflammation Research and Teaching Department, UCL Great Ormond Street Institute of Child Health, London, United Kingdom

**Keywords:** serological survey, RSV, respiratory viruses, respiratory syncytial virus, infant, child

## Abstract

**Background:**

Bronchiolitis caused by respiratory syncytial virus (RSV) is a major cause of mortality and morbidity in infants.

**Aim:**

To describe RSV epidemiology in children in the community in a high-income setting.

**Methods:**

We used stored blood samples from the United Kingdom Born in Bradford cohort study that had been collected at birth, age 1 and 2 years old, tested for IgG RSV postfusion F antibody and linked to questionnaires and primary and hospital care records. We used finite mixture models to classify children as RSV infected/not infected according to their antibody concentrations at age 1 and 2 years. We assessed risk factors for primary RSV infection at each age using Poisson regression models.

**Results:**

The study cohort included 700 children with cord blood samples; 490 had additional blood samples taken at both ages 1 and 2 years old. Of these 490 children, 258 (53%; 95% confidence interval (CI): 48–57%) were first infected with RSV at age 1, 99 of whom (38%; 95% CI: 33–43%) had been in contact with healthcare during peak RSV season (November–January). Having older siblings, birth in October–June and attending formal childcare were associated with risk of RSV infection in infancy. By age 2, a further 164 of 490 children (33%; 95% CI: 29–38%) had been infected.

**Conclusion:**

Over half of children experienced RSV infection in infancy, a further one third had evidence of primary RSV infection by age 2, and one in seven remained seronegative by their second birthday. These findings will inform future analyses to assess the cost-effectiveness of RSV vaccination programmes in high-income settings.

## Introduction

Respiratory syncytial virus (RSV) is a major cause of respiratory morbidity and mortality in young children, accounting for over 33 million episodes of acute lower respiratory tract infection in children under 5 years old per year globally [1,2]. In the United Kingdom (UK), as in many other high-income countries, RSV bronchiolitis is the most common reason for hospitalisation in infants [3]. Infection in early life has been associated with an increased risk of wheeze-related illness throughout childhood [4].

There are currently no RSV vaccines available, although a number of candidates are in clinical trials [5]. Several factors related to the clinical and virological features of RSV challenge the design of an effective vaccination programme, including the very young age at which children are vulnerable to severe RSV morbidity (RSV-related hospital admissions in the UK peak at 1 month of age [6,7]). Further, the short period of immunity induced by natural RSV infection [8], and the seasonal pattern of acute bronchiolitis admissions in temperate countries mean that a successful vaccine will need to induce a stronger immune response than that induced by natural infection [9,10]. In order to determine who should be vaccinated, and when, to effectively protect young children from infection, detailed data on risk of infection according to age and other characteristics, including family structure, are required.

Community-based serological surveys provide crucial data for understanding the epidemiology of RSV, including the proportion of RSV-infected children who are asymptomatic, or who contact healthcare. A community cohort study of children in Kilifi, Kenya has provided insights into RSV dynamics that informed economic modelling of RSV vaccine programmes in low income settings [11,12]. A number of serological surveys of RSV have been carried out in high income settings [13-15]. However, these studies have limited data on risk factors for RSV infection in young children, such as family structure.

We describe the epidemiology of RSV during the first 2 years of life in a cohort of children born in Bradford, UK. Using a unique longitudinal dataset combining serial serological data, parental questionnaires and routinely collected primary care and hospital records, we establish factors associated with maternally-derived antibody concentrations, the proportion of children infected with RSV, their associated healthcare use, and risk factors for RSV infection by age.

## Methods

### Data source

We used data from the Born in Bradford (BiB) cohort study, a longitudinal multi-ethnic birth cohort of 12,453 mothers and 13,773 children recruited in 2007–2011 in Bradford [16]. Upon recruitment, women filled in a baseline questionnaire; cord blood samples were collected at birth. Cohort follow-up is via linkage to electronic health records, including maternity, primary care and hospital records for mothers and children. A subset of mothers whose babies were born on or after 1 March 2008 took part in the BiB Allergy and Infection (ALL-IN) sub-study [17], which collected additional questionnaires about the family and home environment from 2,562 children at age 1 and 2,067 at age 2 years. A total of 5 mL venous blood samples were collected from 1,884 (74%) and 1,623 (79%) children aged 1 and 2 years old, respectively.

### Inclusion criteria

We included all children from the ALL-IN study who had sufficient serial blood samples remaining at the age 1 and 2 years (490 children) [17]. To examine maternally-derived RSV antibody concentrations (that is, antibodies transferred from mother to baby during pregnancy), we additionally tested 210 cord samples from children in the BiB cohort, oversampling children born prematurely (resulting in 700 total cord blood samples). Sample size calculations are presented in Supplementary material 1.

### Serological testing

Blood samples were tested for immunoglobulin G (IgG) antibody against RSV postfusion protein F (post F), and attachment protein G for RSV strands A (Ga) and B (Gb), referred to as IgG post-F, IgG Ga and IgG Gb, respectively, throughout the paper, at the National Institute of Public Health and the Environment (Bilthoven, the Netherlands) using an RSV multiplex immunoassay (details described elsewhere [18]). In short, each of the three RSV antigens were coupled to distinct colour-coded activated carboxylated beads. Serum samples were diluted 1/200 and 1/8,000 in assay buffer containing phosphate-buffered saline with 1% bovine serum albumin and 0.1% Tween 20, incubated with the conjugated beads and subsequently incubated with R-phycoerythrin-labelled goat anti-human IgG antibody. The measurement of the samples was performed using a Bio-Plex 200 in combination with Bio-Plex Manager software version 6.1 (Bio-Rad Laboratories, Hercules, California, United States). The serum IgG antibody concentrations against post-F, Ga and Gb were quantified in arbitrary units/mL by interpolation from a five-parameter logistic curve of an in-house reference serum.

### Outcomes

Our primary outcomes of interest were RSV IgG post-F antibody concentrations, measured in cord blood, and infection status at age < 1 and 1–2 years old derived from IgG post-F levels. Our secondary outcomes were RSV IgG Ga and Gb antibody concentrations. All antibody concentrations were log-transformed for analysis.

### Exposure variables

Study variables are described in Table 1. All analyses included a priori annual quarter of birth (to account for seasonality of RSV, which peaks in early December in the UK), parity, ethnic group and child’s sex. For analyses of maternally-derived antibody concentrations, we additionally included risk factors which could affect placental antibody transfer. For analyses of infection status at age < 1 and 1–2 years, we were additionally interested in indicators of population mixing that we derived from responses to ALL-IN questionnaires at ages 1 and 2 years old, and age at measurement (in months). We derived a binary variable indicating whether the child had had contact with primary or hospital care due to lower or unspecified respiratory tract infection (RTI) during peak RSV season (November–January) before blood measurement (from linked electronic health records, see Supplementary material 1 for a detailed definition).

**Table 1:** Description of variables included in analyses of mean maternally-derived antibody concentrations and risk of primary RSV infection at ages < 1 and 1–2 years, England, 2008–2013

Variable description	Age at measurement	Data source	Variable derivation or categorisation
**Annual quarter of birth**	Birth	Maternity records	January–March; April–June, July–September; October–December
**Parity**	Birth	Maternity records	0; 1; 2; ≥ 3
**Ethnicity**	Birth	Baseline questionnaire	White British; Pakistani origin; other
**Sex**	Birth	Maternity records	Boy/girl
**Gestational age^a^**	Birth	Maternity records	< 35; 35–36; 37–40; ≥ 41 weeks
**Birth weight^a^**	Birth	Maternity records	< 2,500; 2,500–3,499; ≥ 3,500 g
**Gestational diabetes^a^**	Birth	Maternity records	Yes/no
**History of hypertension^a^**	Birth	Maternity records	Yes/no (defined as presence of hypertension, pregnancy-induced hypertension or preeclampsia)
**Household size^b^**	1 and 2 years (2 variables)	ALL-IN questionnaires at 1 and 2 year	2–4 or ≥ 5 household members
**Sharing a bedroom^b^**	0–5 months, 6–11 months, 12–23 months (3 variables)	ALL-IN questionnaires at 1 and 2 year	Yes/no
**Attending formal childcare^b^**	0–11 months, 12–23 months (2 variables)	ALL-IN questionnaires at 1 and 2 year	Yes/no (defined as attending nursery or being cared for by a childminder/nanny)
**Attending mother–baby activities^b^**	6–11 months, 12–23 month (2 variables)	ALL-IN questionnaires at 1 and 2 year	Rarely/once a week or more often
**Age at blood sample measurement^b^**	1 and 2 years (2 variables)	ALL-IN questionnaires at 1 and 2 year	Age in months
**RTI-related contact with healthcare during RSV season before blood measurement^b^**	Any contact for < 1 and 1–2 years olds	Linked primary care and hospital records	Yes/no

ALL-IN: Allergy and Infection sub-study; RSV: respiratory syncytial virus; RTI: respiratory tract infection.

^a^ Indicates variables used only for analyses of mean maternally-derived antibody concentrations.

^b^ Indicates variables used only for analyses of risk of RSV infection.

Unless otherwise specified (footnotes a and b above), variables were used in both sets of analyses.

### Statistical analyses

We derived the number and proportion of children in our study and in the full ALL-IN cohort by risk factors at birth, age 1 and 2 years old.

#### Maternally-derived respiratory syncytial virus antibody concentrations

We derived mean maternally-derived log_e_ RSV IgG post-F antibody concentrations according to each risk factor category (listed in Table 1). We fitted a log-linear regression model to identify exposures associated with higher mean maternally-derived RSV IgG post-F levels. We determined factors associated with log_e_ RSV IgG post-F antibody concentrations in univariate analyses using analysis of variance (ANOVA). We included all risk factors significantly associated with placental antibody transfer in univariate analyses in the multivariable model (where p < 0.05); we excluded those that were not statistically significant in the mutually adjusted model from the final multivariable model according to Wald’s test (where p > 0.05).

#### Primary respiratory syncytial virus infection at ages 1 and 2 years old

We applied a two-stage modelling strategy to determine risk factors for RSV infection. First, we used finite mixture models (FMM) to classify children as RSV infected at age < 1 year and 1–2 years respectively according to their log_e_ RSV IgG post-F levels at age 1 and 2 years old. FMM is a well-established data driven method for classifying past exposure to infection [19,20]. FMM assume that the observed sample comes from distinct unobserved subpopulations. We expected two subpopulations (infected/not-infected). The model was as follows:

$$f\left(\log_{e}\left(\text{RSV IgG post-F}\right)\right)=\pi_{1}\times f_{1}\left(\log_{e}\left(\text{RSV IgG post-F}\right)\right)+\pi_{2}\times f_{2}\left(\log_{e}\ln\left(\text{RSV IgG post-F}\right)\right)$$

Where (*π_1_,π_2_*) are probabilities of belonging to each subpopulation, and (*f_1_,f_2_*) are density functions for antibody concentrations in each subpopulation. For antibody concentrations at age 2, we allowed (*π_1_,π_2_*) to depend on observed antibody concentrations at age 1. Details of FMM selection are described in Supplementary material 1.

For each child, we then derived a binary indicator of serological evidence of past RSV infection as:

$${RSV\;Infection}_{i}=\left\{\begin{array}{c} 1\;if\;p_{i}\geq 0.5\\ 0\;otherwise \end{array}\right.$$

Where *p_i_* is the posterior probability of infection from FMM for child *i*. Throughout the paper we refer to presence of serological evidence of past RSV infection from the FMM as ‘RSV infection’. We calculated the number and proportion of children who acquired primary RSV infection aged < 1 year and 1–2 years old. We derived the proportion of infected (seropositive) and seronegative children who had RTI-related contact with healthcare during peak RSV season. We compared the distribution of risk factors in RSV-infected and never-infected children by age.

We estimated risk ratios (RR) for primary RSV infection at age < 1 and 1–2 years old using Poisson regression models with robust error variances calculated using sandwich estimators [21]. We decided a priori to include child’s age at blood sample measurement and annual quarter of birth, sex and ethnicity in the models. We added all indicators of population mixing to the model (listed in Table 1). Then we removed those that were not statistically significant according to Wald’s test (where p > 0.05).

#### Sensitivity analyses

Infections in infants aged < 6 months old may not be detectable using serology, as young infants do not mount an antibody response to infection (or their response might be ‘masked’ by maternally-derived antibodies) [22-24]. We therefore repeated all analyses after re-classifying children who had RTI-related contact with healthcare aged < 6 months during RSV season (October–February) and during peak RSV season (November–January) as infected at age < 1 year.

To account for possible misclassification of infection status arising from using a cut-off for the posterior probability of infection from the FMM, *p_i_*  , we simulated the infection status for each child *i* as a random draw from Bernoulli distribution with *p_i_* taken as the probability of success. We then re-calculated RR for primary infection. We repeated this 50 times and pooled estimates of RRs using Rubin’s rules for the parameter estimates and variance-covariance matrix [25]. We repeated all analyses using log_e_ RSV IgG Ga and Gb. All analyses were carried out using Stata version 15.

### Ethical statement

Parents in BIB and ALL-IN studies have given informed consent for use of data and stored blood samples for research studies. The ALL-IN study has been approved by the Bradford Research Ethics Committee, reference number 08/H1302/21.

## Results

**Figure 1:**
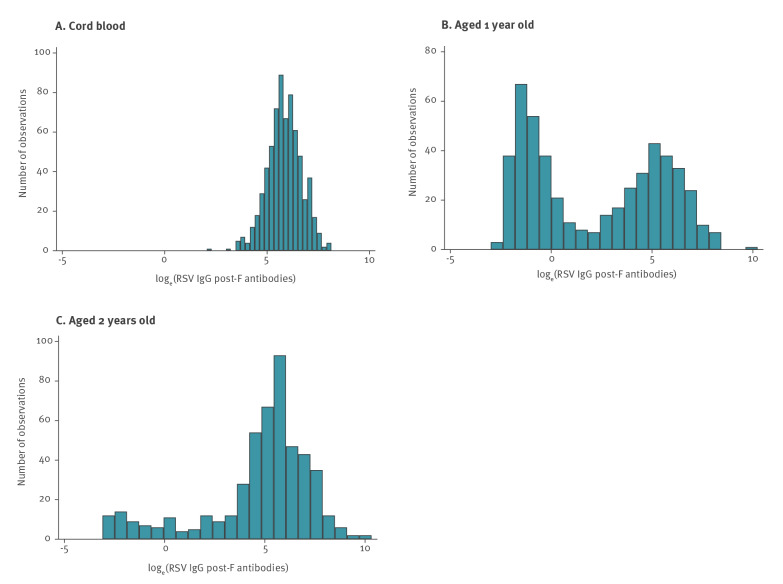
Distribution of log_e_ RSV IgG post-F antibodies (A) at birth (n = 700 cord blood samples), (B) age 1 (n = 490 children) and (C) 2 years old (n = 490 children), England, 2008–2013

The cohort included 700 children with cord blood samples, of whom 683 (98%) had complete information on all risk factors included in the final analyses of maternally-derived antibody concentrations. A total of 490 children had blood samples measured at both age 1 and 2 years old, of whom 477 (97%) had complete information on risk factors included in the final analyses of risk factors for primary RSV infection. Over half of children (55%; 384/700) were of Pakistani origin compared to 49% (1,251/2,562) in the ALL-IN study (Supplementary Table S3). Reflecting oversampling of preterm babies, 11% (76/700) of the children in the cohort were born prematurely vs 5.8% (149/2,562) in the ALL-IN study and a third were first-born (227/700) compared to 36%, 920/2,562 in the ALL-IN study. Mean age at first ALL-IN questionnaire was 13.1 months (95% confidence interval (CI): 13.0–13.2 months, Supplementary Table S4), and at second questionnaire was 25.6 months (95% CI: 25.5–25.7 months). Maternally-derived antibodies showed an approximately normal distribution; at age 1 antibody concentrations were equally distributed around two peaks; at 2 years old, the majority of observations were centred around the higher peak (Figure 1).

IgG post-F: immunoglobulin G antibody against RSV postfusion protein F; log_e_: natural logarithm (base e); RSV: respiratory syncytial virus.

IgG post-F concentrations were quantified in arbitrary units/mL.

### Maternally-derived respiratory syncytial virus antibody concentrations

Gestational age, parity and annual quarter of birth were significantly associated with maternally-derived RSV antibody concentrations (see Supplementary Table S5 for detailed results). Children born at < 35 gestational weeks had 35% lower, and children born at ≥ 41 weeks had 19% higher, mean maternally-derived IgG post-F levels compared with children born at 37–40 weeks (Table 2). Presence of 1–2 older siblings compared with no older siblings was associated with 18–20% higher mean maternally-derived antibody concentrations, and ≥ 3 siblings with 8% higher levels. Children born outside October–December had 19–22% higher mean maternally-derived antibody concentrations than children born during these months.

**Table 2:** Risk factors associated with increase in mean maternally-derived RSV IgG post-F antibody levels, England, 2008–2013 (n = 683 children)^a^

Risk factor	Distribution of children included in the analyses by each risk factor (n = 683)^b^		Adjusted ratios of geometric mean maternally-derived IgG post-F antibody levels (95% CI)^c,d^
	N	%	
**Gestational age (weeks)**			
** < 35**	29	4.2	0.65 (0.48–0.89)
**35–36**	47	6.9	0.90 (0.70–1.16)
**37–40 (baseline)**	480	70.3	1 (baseline)
**≥ 41**	127	18.6	1.19 (1.01–1.39)
**Parity**			
**0 (baseline)**	227	33.2	1 (baseline)
**1**	203	29.7	1.20 (1.03–1.40)
**2**	124	18.2	1.18 (0.99–1.42)
**≥ 3**	129	18.9	1.08 (0.90–1.30)
**Time of birth**			
**Jan–Mar**	186	27.2	1.19 (1.00–1.42)
**Apr–Jun**	165	24.2	1.22 (1.02–1.46)
**Jul–Sep**	159	23.3	1.22 (1.03–1.46)
**Oct–Dec (baseline)**	173	25.3	1 (baseline)

CI: confidence interval; IgG post-F: immunoglobulin G antibody against RSV postfusion protein F; RSV: respiratory syncytial virus.

^a^ The cohort included 700 children with cord blood samples, of whom 683 had complete information on all risk factors included in the final analyses of maternally-derived antibody concentrations.

^b^ The number and percentages of children, per risk factor category included in the final model, are presented.

^c^ The exponentiated results from the log_e_-linear model (mutually adjusted for all variables in the table), reflecting proportional change in maternally-derived IgG post-F levels for each risk factor category relative to the baseline are shown.

^d^ The mean maternally-derived RSV IgG post-F antibody concentration at baseline (gestational age of 37–40 weeks, parity of 0 and birth in October–December) was 268 arbitrary units/mL (95% CI: 229–314 arbitrary units/mL).

### Primary respiratory syncytial virus infection at ages 1 and 2 years old

**Figure 2:**
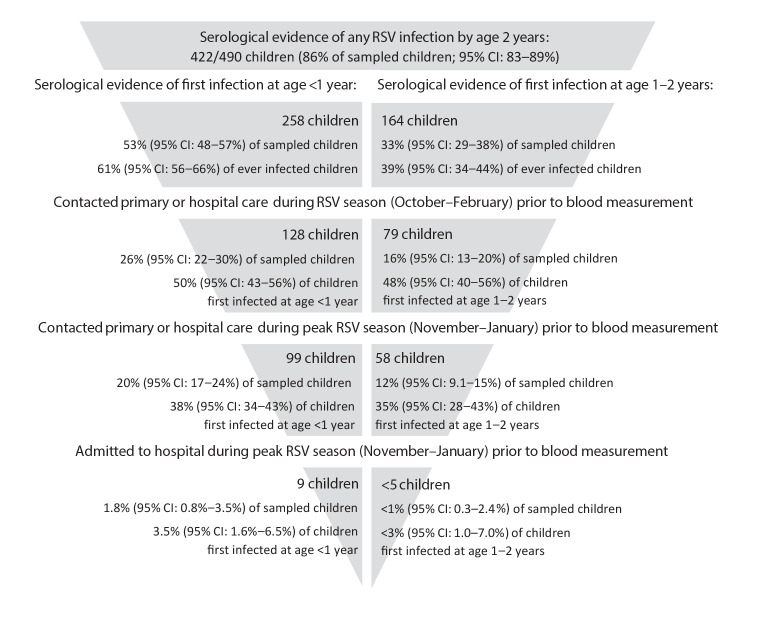
Distribution of primary RSV infections in children by age at first infection and severity of infection (as indicated by contact with healthcare), England, 2008–2013 (n = 490 children)

By age 1 year, 258/490 children (53%; 95% CI: 48–57%, Figure 2) had serological evidence of past RSV infection, 99 of whom had RTI-related contact with primary or hospital care in November–January (38% of RSV infected children; 95% CI: 34–43%); nine had had an RTI-related hospitalisation. Of 232 children who were RSV seronegative at age 1, 48 (21%; 95% CI: 16–26%) had RTI-related contact with healthcare in November–January (of whom 29 were aged < 6 months old). A further 164 of 490 children had serological evidence of primary RSV infection at age 1–2 years old (33%; 95% CI: 29–38%), 58 of whom had RTI-related contact with primary or hospital care in November–January (35% of children first infected at age 1–2; 95% CI: 28–43%). A proportion of 68/490 children (14%; 95% CI: 11–17%) had no evidence of RSV infection by age 2, of whom 13 (19%; 95% CI: 11–30%) had RTI-related contact with primary or hospital care in November–January.

CI: confidence interval; RSV: respiratory syncytial virus.

Left-hand side shows the number and proportion of children first infected aged < 1 year old, right-hand side show the proportion of children first infected aged 1–2 years old (that is, they were never infected at age < 1). We present the number of children who contacted healthcare using a broad definition of RSV season (October–February) and peak RSV season (November–January).

Factors associated with an increased risk of RSV infection aged < 1 year old were annual quarter of birth, number of older siblings, and attending formal childcare (Table 3, see Supplementary Table S6 for detailed analyses). Children with one older sibling had 49% higher risk of infection in infancy relative to children with no older siblings. Having two, or more than two, siblings increased the risk of infection by 22% and 38%, respectively, compared to no siblings. Children who attended formal childcare had 36% higher risk of infection compared with children who did not attend formal childcare. The risk of infection was 57–63% higher for children born during or after RSV season (i.e. in October–June). The risk of primary infection at age 1–2 years old was greater for children with siblings compared with children with no siblings, and for children born during the RSV season (October–March, Table 3).

**Table 3:** Adjusted risk ratios for primary RSV infection at age 1 and 2 years old by risk factor, England, 2008–2013 (n = 477)^a^

Risk factor	Adjusted risk ratios for primary RSV infection at age < 1 year old (95% CI)^b^	Adjusted risk ratios for primary RSV infection at age 1–2 years old (95% CI)^b^
Number of children included in the model	477	229
Number of infected children	248	162
Sex		
Male (baseline)	1 (baseline)	1 (baseline)
Female	1.07 (0.91–1.27)	1.12 (0.95–1.32)
Ethnic group		
White British (baseline)	1 (baseline)	1 (baseline)
Pakistani origins	1.23 (0.98–1.55)	0.88 (0.73–1.05)
Other	1.27 (0.95–1.69)	1.01 (0.80–1.28)
Annual quarter of birth		
Jul–Sep (baseline)	1 (baseline)	1 (baseline)
Oct–Dec	1.57 (1.19–2.08)	1.23 (1.02–1.48)
Jan–Mar	1.57 (1.18–2.09)	1.12 (0.89–1.41)
Apr–Jun	1.63 (1.22–2.18)	0.77 (0.54–1.09)
Parity		
0 (baseline)	1 (baseline)	1 (baseline)
1	1.49 (1.18–1.87)	1.27 (1.04–1.56)
2	1.22 (0.93–1.60)	1.20 (0.93–1.56)
≥ 3	1.38 (1.06–1.79)	1.34 (1.05–1.70)
Attending any formal childcare		
No (baseline)	1 (baseline)	Not included in the final model
Yes	1.36 (1.09–1.69)	
Age at measurement^c^	1.08 (1.00–1.16)	1.02 (0.96–1.08)

CI: confidence interval; RSV: respiratory syncytial virus.

^a^ Of 490 children whose blood samples were tested for immunoglobulin G antibody against RSV postfusion protein F, 477 (97%) had complete information for the analysis of risk factors for primary RSV infection at age <1 year old. Of these, 229 had not experienced an infection in infancy and were included for the analysis of risk factors for primary RSV infection at age 1–2 years.

^b^ Risk ratios for primary RSV infection by each risk factor were estimated from Poisson regression models with robust error variances calculated using sandwich estimator.

^c^ Age at measurement was centred around 12 months for risk ratios for primary RSV infection at age < 1 year, and around 24 months for risk ratios for primary RSV infection at age 1–2 years.

### Sensitivity analyses

We identified an additional 40 children who had RTI-related contact with healthcare in October–February aged < 6 months. After re-classifying these children as infected in infancy, we estimated that 61% (299/490; 95% CI: 57–65%) of children were RSV-infected aged < 1 year, 28% (137/490; 95% CI: 24–32%) were newly infected aged 1–2 years old and 11% (54/490; 95% CI: 8–14%) had no evidence of RSV infection by their second birthday. RRs did not change substantially (Supplementary Tables S7 and S8), apart from the effect of annual quarter of birth on risk of primary RSV infection in infancy, which reduced substantially for all annual quarters compared with children born in July–September. Sensitivity analyses included a higher number of RSV infections in the baseline category (born in July–September) compared with the main analyses, leading to a smaller, non-significant effect of annual quarter of birth. The pooled estimates from 50 simulated outcomes were consistent with results from the main analysis.

Analyses of maternally-derived IgG Ga and Gb showed similar results to those for IgG post-F (Supplementary Table S9). The indicator of RSV infection in infancy derived using each IgG post-F, Ga and Gb agreed for 58% (284/490) of children, 32% (156/490) showed agreement between IgG post-F and either Ga or Gb antibodies (Supplementary Table S10). Since IgG Ga and Gb antibodies are less immunogenic than IgG post-F [18], we did not re-calculate RRs using RSV infection indicators from these models (see Supplementary material**2 for details).

## Discussion

By the end of their first year of life, 53% of children experienced at least one RSV infection, 38% of whom had RTI-related contact with healthcare during peak RSV season. A further 33% of children experienced their first RSV infection aged 1–2 years old, 35% of whom had RTI-related contact with primary or hospital care during the RSV season. One in seven children had no evidence of RSV infection by age 2. Having older siblings, attending formal childcare and birth between October and June were predictive of RSV infection at age < 1. Having older siblings contributed to increased risk of RSV infection between 1 and 2 years. Children born at later gestational ages, in families with older siblings and after the first half of the RSV season (October–December) had higher mean maternally-derived antibody concentrations.

### Strengths and limitations

We used a unique collection of longitudinal blood samples in children up to 2 years of age, linked to rich questionnaire data with information on risk factors in the child’s family and home environment. Routinely collected electronic health records from primary care and hospitals provided us with indicators for more severe symptomatic RTIs leading to healthcare contact. Infection status was derived from antibody data using robust statistical methods. Instead of costly and time-consuming data collection for a de-novo serological survey, we re-used previously collected data, minimising the costs of the study, and the burden of data collection and blood sampling on families and children.

A limitation of our study is that we were not able to validate infection status derived from FMM against confirmed RSV infections, since nasopharyngeal aspirates were not collected for the ALL-IN study. This would be useful for indicating infections in infants aged < 6 months, who are less likely to exhibit serological response to infection [22-24]. Children < 6 months old have the highest risk of developing RSV-related lower RTI [26], and their parents are therefore most likely to contact healthcare for their children. Therefore, our sensitivity analyses including children who had RTI-related contact with healthcare at younger ages likely accounted for most of the early infections and showed consistent results. Further, we were not able to assess how many children were re-infected during their second year of life. Ideally, repeated swab sampling during the first 2 years of life is required to estimate the rate of reinfection.

Our study focussed on one geographical area in the UK, with a multi-ethnic, urban population. Therefore, our results may be predominantly applicable to similar settings in high income countries. Our methods, however, could be applied to residual blood samples from other cohort studies where blood samples have been collected at similar intervals for comparison.

### Implications

We found that over half of children in this UK study experienced at least one RSV infection in infancy, and a further third of children had evidence of primary RSV infection between the ages of 1 and 2 years. Our estimates of the incidence of RSV in the community were comparable with a serological survey from Kilifi (52% of children were RSV-seropositive at age 12–15 months and 83% at age 18–24 months in 2007–2010) [27], higher than in a Finnish study (37% had evidence of infection by age 1, 68% by age 2 in 2009–2013) [15], and lower than the often quoted study from 1986 carried out in Houston, US [14], in which 68% of 125 infants and 91/92 children aged 1–2 years had been infected with RSV. In contrast, we found that one in seven children remained uninfected by the age of 2. These differences could reflect differences in study settings and serological testing methods and performance (e.g. one study is now over 35 years old). Since a substantial proportion of children experienced primary infection during their second year of life, a vaccination programme for older infants with a catch-up school programme will be important in reducing the community burden of RSV [11]. A third of children with primary infection in the second year of life were in contact with primary care services. RSV infections in this older age group therefore contribute considerably to the RSV-related burden on health services, and should be taken into account in modelling studies evaluating RSV vaccination programmes.

The presence of older siblings in the household was the risk factor consistently predictive of the risk of primary RSV infection both in infancy and aged 2 years old, even after adjusting for exposure to formal childcare environments. Older siblings, in particular school-age older siblings, are the most likely source of RSV infections in households [11,13,28], and contribute to increased risk of RSV-related hospitalisation in young children [6]. Therefore, preventive strategies to reduce early infections in young children, whether public health campaigns or vaccination strategies, need to include reducing the risk of infection posed by older siblings.

Maternally-derived antibody concentrations were 20% lower for children born in the first half of RSV season (October–December) than in other months, but we did not observe a strong seasonal pattern as that reported for neutralising antibodies, with increases in mean RSV antibody concentrations between January–June and decreases thereafter [29]. We found that levels of maternally-derived antibodies were lower for premature babies, reflecting reduced transplacental antibody transfer at earlier gestations [30,31], and higher for mothers with older children, who are likely to introduce RSV infections into households [11,13,28]. Since the majority of RSV-related hospitalisations occur in term babies [6,7], our findings suggest that pregnant women with older children are a particularly important group for targeting via maternal vaccination programmes.

## Conclusions

We have shown that 53% of children in England are infected with RSV during the first year of life, and 14% remain uninfected by age 2. Between birth and 2 years old, the proportion of RSV infected children who seek primary or secondary healthcare does not decrease with age. Our study, based on secondary analyses of blood samples and linked data, present a time-efficient and cheap method for carrying out serological surveys to determine the community burden of disease due to RSV in young children.

## Supplementary Data

7290000 Supplementary Material
