# Supplementary material for: Respiratory syncytial virus in young children: community cohort study integrating serological surveys, questionnaire and electronic health records, Born in Bradford cohort, England, 2008 to 2013
Source: Euro Surveill. 2021 Feb 11;26(6):2000023. doi: 10.2807/1560-7917.ES.2021.26.6.2000023 (PMC7879500; doi:10.2807/1560-7917.ES.2021.26.6.2000023)
Supplement: Supplementary Material [file 2000023_Supplementary_material.pdf]

Supplementary Material

This supplementary material is hosted by *Eurosurveillance* as supporting information alongside the article “Respiratory syncytial virus in young children: community cohort study integrating serological surveys, questionnaire and electronic health records, Born in Bradford cohort, England, 2008 to 2013”, on behalf of the authors, who remain responsible for the accuracy and appropriateness of the content. The same standards for ethics, copyright, attributions and permissions as for the article apply. Supplements are not edited by Eurosurveillance and the journal is not responsible for the maintenance of any links or email addresses provided therein.

Supplementary Materials 1: Additional description of methods

1.1 Sample size calculations

We tested 490 left-over blood samples collected from the Allergy and Infection (ALL-IN) study at age 1 and 2 years old. We included all children who had sufficient serial samples remaining from the ALL-IN study at the two ages.[1] To determine a change in RSV antibody concentrations between age 1 and 2 years, we estimated a sample size of 276 children, based on detecting an increase in respiratory syncytial virus (RSV) seroprevalence from 65% to 80% between 1 and 2 years old with 80% power.[2]

Assuming a difference in $\log_2$ immunoglobulin G antibody against RSV postfusion protein F levels of 15% or greater[3] we required 700 cord blood samples to detect a difference among children born preterm (<37 weeks gestation) and term-born children with 80% power. To examine maternal RSV antibody concentrations, we therefore tested an additional 210 cord samples from children in the original Born in Bradford (BiB) cohort. Among the 210 cord samples, we oversampled children born prematurely.

1.2 Deriving indicator of contact with healthcare

We used electronic health records (hospital admission, primary care and primary care prescribing records) to derive indicators of contact with healthcare due to respiratory tract infection (RTI) during peak RSV season as a proxy for likely symptomatic infection.[5] Children were indicated as having RTI-related contact with healthcare if any of the criteria listed below were met during peak RSV season (defined as 1st November – 31st January).

We used the same method to indicate children who had RTI-related contact with healthcare aged <6 months during RSV season (defined as 1st October – 28th/29th February) for sensitivity analyses 1 and peak RSV season (1st November – 31st January) for sensitivity analyses 2.

1.2.1 Hospital records

We derived an indicator of hospital contact via linkage to Bradford Royal Infirmary (the main hospital serving the city of Bradford) electronic hospital records. Records were deterministically linked to children in the cohort using National Health Service (NHS) number. Diagnostic information was recorded using International Classification of Diseases version 10 (ICD-10) codes.[6]

To identify RSV-related hospital admissions, we flagged hospital admissions where any of the diagnoses recorded during the admission included either of the following of the ICD-10 codes[7]:

- J21: Bronchiolitis
- J12.1: Respiratory syncytial virus pneumonia
- J20.5: Acute bronchitis due to respiratory syncytial virus
- B97.4: Respiratory syncytial virus as the cause of diseases classified to other chapters
- B34.9: Viral infection, unspecified and R06.2: Wheezing (recorded together during the same admission)

1.2.2 Primary care records

Primary care records were obtained through linkage to SystmOne, a database covering clinical codes and prescriptions recorded during appointments by General Practitioners (GP) and maintained by The Phoenix Partnership (TPP).[6,8] Records were deterministically linked to BiB participants using their

NHS number, surname, date of birth and sex. 97% of 13,857 children in BiB were linked to a primary care record.[9]

Clinical information was recorded using Read medical codes version 3 (CTV3).[9] To identify RSV-related contact with primary care, we included 77 CTV3 Term ID codes, which we translated to 129 CTV3 concept IDs (listed in appendix table 1) for symptoms such as: fever, rigor, cough, and codes likely to indicate RSV infection (RSV, respiratory tract infection, bronchitis, bronchiolitis, chest infection, pneumonia).

Prescriptions issued in primary care are coded using sections of the British National Formulary (BNF).[10] We extracted prescriptions from BNF chapter 5.1 and indicated prescriptions for amoxicillin if drug name included “amoxicillin”. We focus on amoxicillin as it is indicated for community acquired pneumonia, and it is the most commonly prescribed antibiotic for respiratory tract infections.[11] Our previous research has demonstrated that the peak timing of amoxicillin prescribing coincides with the peak in RSV circulation in the UK.[12]

Supplementary Table S1 – Read codes used to identify RSV-related contacts with primary care

CTV3 Term ID	description of CVT3 Term ID	CTV3 Concept ID
Y100B	Acute respiratory infections	.H1..
Y100B	Acute respiratory infections	H0...
Y100B	Acute respiratory infections	XE0Yl
Y100C	Acute respiratory infection NOS	H0z..
Y100C	Acute respiratory infection NOS	XE0Yv
Y100C	Acute respiratory infection NOS	H05y.
Y100n	Acute lower respiratory tract infection	.H163
Y100n	Acute lower respiratory tract infection	H062.
Y100n	Acute lower respiratory tract infection	H06z1
Y100n	Acute lower respiratory tract infection	XE0Xt
Y100o	Chest infection	.H163
Y100o	Chest infection	.H6ZA
Y100o	Chest infection	H062.
Y100o	Chest infection	H06z0
Y100o	Chest infection	XE0Xt
Y100q	Chest infection NOS	.H6ZA
Y100q	Chest infection NOS	H06z0
Y100q	Chest infection NOS	XE0Xs
Y100r	Acute bronchitis and bronchiolitis	.H16.
Y100r	Acute bronchitis and bronchiolitis	H06..
Y100s	Acute bronchitis or bronchiolitis NOS	H06z.
Y100t	Acute tracheobronchitis	H5yy.
Y100t	Acute tracheobronchitis	.H161
Y100t	Acute tracheobronchitis	H0605
Y100T	Respiratory infection NOS	XE0Yt
Y100u	Tracheobronchitis NOS	H300.
Y100v	Acute bronchitis	.H161
Y100v	Acute bronchitis	H060.
Y100v	Acute bronchitis	XE0Xr
Y100v	Acute bronchitis	XE0Yt
Y100w	Acute wheezy bronchitis	H060.
Y100w	Acute wheezy bronchitis	XM1QX
Y100y	Chest infection - unspecified bronchitis	H30..
Y100y	Chest infection - unspecified bronchitis	X1006
Y100z	Acute fibrinous bronchitis	H0600
Y1010	Acute membranous bronchitis	H0601
Y1011	Acute pseudomembranous bronchitis	H0602
Y1012	Acute purulent bronchitis	H0603
Y1013	Acute croupous bronchitis	H0604
Y1014	Subacute bronchitis unspecified	H060v
Y1015	Acute bronchitis NOS	.H16Z
Y1015	Acute bronchitis NOS	H060z
Y101G	Acute viral bronchitis unspecified	H060w
Y101H	Acute bronchiolitis	.H162
Y101H	Acute bronchiolitis	H061.
Y101I	Acute capillary bronchiolitis	H0610
Y101K	Acute bronchiolitis with bronchospasm	H0612
Y101L	Acute exudative bronchiolitis	H0613
Y101N	Acute bronchiolitis NOS	H061z
Y102y	Viral pneumonia	.H31.
Y102y	Viral pneumonia	H20..
Y102y	Viral pneumonia	XE0YG

Y102z	Chest infection - viral pneumonia	H20..
Y1031	Pneumonia due to respiratory syncytial virus	H201.
Y1033	Viral pneumonia NEC	H20y.
Y1034	Viral pneumonia NOS	H20z.
Y106m	Bronchitis unspecified	H30..
Y106m	Bronchitis unspecified	XE0YL
Y106n	Bronchitis NOS	.H6Z1
Y106n	Bronchitis NOS	H30z.
Y108i	Recurrent wheezy bronchitis	H3310
Y108j	Wheezy bronchitis	H3311
Y108j	Wheezy bronchitis	XE0YU
Y7DUa	Fever with sweating	.1653
Y7DUa	Fever with sweating	1653.
Y7DUo	Pyrexia symptoms	.165.
Y7DUo	Pyrexia symptoms	165..
Y7DUo	Pyrexia symptoms	X76Dk
Y7DUp	Fever symptoms	.165.
Y7DUp	Fever symptoms	165..
Y7DUp	Fever symptoms	X76Dl
Y7DUQ	Temperature symptom NOS	.165Z
Y7DUQ	Temperature symptom NOS	165Z.
Y7DUR	Temperature symptoms	.165.
Y7DUR	Temperature symptoms	165..
Y7DUR	Temperature symptoms	XE2ti
Y7DUY	Feels hot/feverish	.1652
Y7DUY	Feels hot/feverish	1652.
Y7Dva	Productive cough-yellow sputum	.1715
Y7Dva	Productive cough-yellow sputum	1715.
Y7Dvk	Sputum - symptom	.171.
Y7Dvk	Sputum - symptom	171..
Y7Dvkf	Sputum - symptom	X76I3
Y7DvP	Cough	.171.
Y7DvP	Cough	171..
Y7DvP	Cough	XE0qn
Y7DvR	Cough symptom NOS	.171Z
Y7DvR	Cough symptom NOS	171Z.
Y7DvU	Dry cough	.1712
Y7DvU	Dry cough	1712.
Y7DvX	Productive cough NOS	.1716
Y7DvX	Productive cough NOS	1716.
Y7DvX	Productive cough NOS	XE0qo
Y7DvY	Productive cough -green sputum	.1714
Y7DvY	Productive cough -green sputum	1714.
Y7DvZ	Productive cough -clear sputum	.1713
Y7DvZ	Productive cough -clear sputum	1713.
Y7DWV	Having rigors	.1654
Y7DWV	Having rigors	1654.
Y7DWV	Having rigors	XE0qf
Y7DWW	Rigor - symptom	.1654
Y7DWW	Rigor - symptom	1654.
Y7DWW	Rigor - symptom	X76EQ
Ya1Cl	Respiratory syncytial virus infection	A79A.
Ya1Cl	Respiratory syncytial virus infection	Xa0BK
Yaaft	Recurrent chest infection	H06z2

Yaaft	Recurrent chest infection	XaBM8
Yah6P	RSV antigen level	.43dS
Yah6P	RSV antigen level	43dS.
Yah6P	RSV antigen level	XaFvJ
Yakn7	Feverish cold	.1656
Yakn7	Feverish cold	1656.
Yakn7	Feverish cold	XaIO0
Yakn8	Cough with fever	.171F
Yakn8	Cough with fever	171F.
Yakn8	Cough with fever	XaIO1
YaoZp	RSV nucleic acid detection	.43jk
YaoZp	RSV nucleic acid detection	43jk.
YaoZp	RSV nucleic acid detection	XaLTa
Yas5I	Respiratory syncytial virus A detected	.4JU9
Yas5I	Respiratory syncytial virus A detected	4JU9.
Yas5I	Respiratory syncytial virus A detected	XaPOa
Yas5K	Respiratory syncytial virus B detected	.4JUA
Yas5K	Respiratory syncytial virus B detected	4JUA.
Yas5K	Respiratory syncytial virus B detected	XaPOb
Yas5M	Respiratory syncytial virus untyped strain detected	.4JUB
Yas5M	Respiratory syncytial virus untyped strain detected	4JUB.
Yas5M	Respiratory syncytial virus untyped strain detected	XaPOc
YaY9i	Laryngotracheobronchitis	H301.
YaYAg	Chest cold	H07..
YM0Qw	O/E - stridor present	.2DE2
YM0Qw	O/E - stridor present	2DE2.
YM10a	[D]Cough	.R62.
YM10a	[D]Cough	R062.
YM1ir	C/O - cough	.171.
YM1ir	C/O - cough	171..
YM1ir	C/O - cough	XM0Ch
YM9gh	Coughing up phlegm	1716.
YM9gi	Chesty cough	.1719
YM9gi	Chesty cough	1719.
YM9gj	Bronchial cough	.1719
YM9gj	Bronchial cough	1719.
YM9i7	Respiratory tract infection	H06z1
YM9i7	Respiratory tract infection	XM1QW
YMAit	Acute bronchiolitis due to respiratory syncytial virus	Hyu08
YMAIt	[X]Other viral pneumonia	H0615
YMAJ3	[X]Other acute lower respiratory infections	Hyu1.
YMAJ4	[X]Acute bronchitis due to other specified organisms	Hyu10
YMK6I	Acute bronchitis due to respiratory syncytial virus	H060D
YMK6K	Lower resp tract infection	H06z1
YSOit	Respiratory syncytial virus antigen assay	.43dn
YSOit	Respiratory syncytial virus antigen assay	43dn.
YSOit	Respiratory syncytial virus antigen assay	XSEzR

RSV= respiratory syncytial virus

1.3 Deriving RSV infection status using finite mixture models

We applied finite mixture models (FMM) to the log RSV IgG post-F levels at age 1 and 2 years old to classify children as RSV infected at age <1 year and 1-2 years respectively according to their antibody concentration levels. *A priori* we decided to fit a model with 2 classes (infected / not-infected). The model was as follows:

$$f(\ln(\text{RSV IgG post-F})) = \pi_1 \times f_1(\ln(\text{RSV IgG post-F})) + \pi_2 \times f_2(\ln(\text{RSV IgG post-F}))$$

where π_1 and π_2 are the probabilities of observation belonging to each class, f_1 and f_2 are conditional probability density functions for observed antibody concentrations in each class.

For antibody levels at age 2 years old, we considered a model with no covariates for class probabilities π_1, π_2 (model 1), and a model allowing the probabilities of infection π_1, π_2 to depend on observed antibody concentrations at age 1 (model 2). We considered this covariate in the model as IgG post-F concentrations were likely to remain at a higher level following infection at age 1-2 years in children who were first infected in infancy. We compared latent class marginal mean IgG post-F levels, marginal posterior probabilities and Akaike's Information Criterion (AIC) for the two models. We used model 2 in the final analyses as it had lower AIC (supplementary table 2).

Supplementary Table S2 - Model selection for finite mixture model at age 2 years old.

	**Latent class marginal mean log IgG post-F levels (95% CI)**	**Latent class marginal probability (95% CI)**	**AIC**
**Model (1) – no covariates**			2,141
group 1	-0.74 (-1.32, -0.16)	15.6% (12.2%, 19.9%)	
group 2	5.71 (5.55, 5.86)	84.4% (80.1%, 87.8%)	
**Model (2) – include IgG at age 1 year**			2,068
group 1	-0.93 (-1.38, -0.48)	14.7% (11.8%, 18.2%)	
group 2	5.72 (5.56, 5.88)	85.3% (81.8%, 88.2%)	

AIC = Akaike's Information Criterion, CI=confidence interval, IgG post-F = immunoglobulin G antibody against postfusion protein F.

The two latent classes (RSV infected vs not infected) were well defined – 475 (97%) children at age 1 and 474 (97%) children at age 2 a posterior probability of infection either <10% or >90% (supplementary figure 1). 5 children were classified as not infected at age 2 years old, but infected at age 1 year old.

Supplementary Figure S1 – Posterior probabilities of RSV infection at age 1 and 2 years old according to IgG post-F antibodies

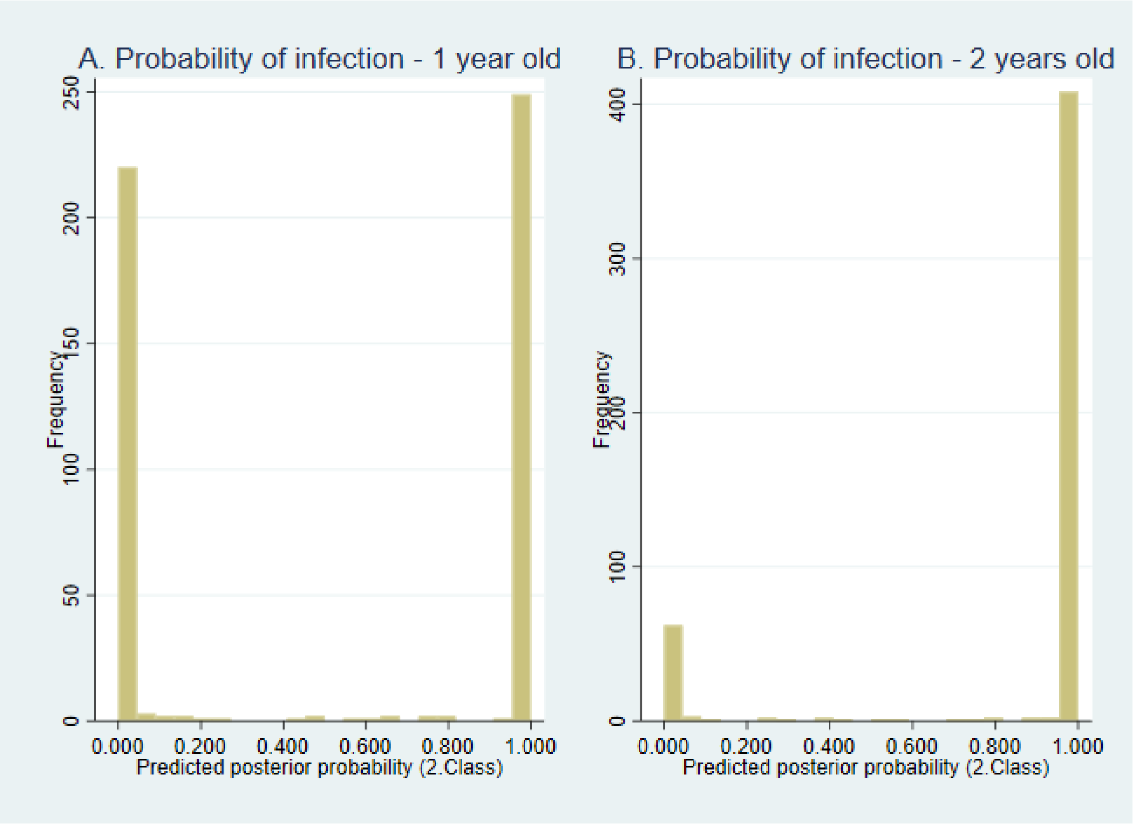

IgG post-F = immunoglobulin G antibody against postfusion protein F, RSV= respiratory syncytial virus

Appendix 2:

Supplementary Materials 2: Additional results

2.1 Comparison of study cohort and the ALL-IN study participants

Supplementary Table S3 – Baseline characteristics of children with cord blood samples, sub-sample of children with additional blood samples at age 1 and 2 years old compared to all participants of the ALL-IN study.

Risk factor category	ALL-IN children n=2562	Sampled children with tested cord blood samples* n=700		Children with longitudinal blood measurements n=490	
	N (%)	N (%)	p-value	N (%)	p-value
**Gestational age (weeks)**			<0.01		0.04
<35	69 (2.7%)	29 (4.1%)		4 (0.8%)	
35-36	80 (3.1%)	47 (6.7%)		10 (2.0%)	
37-40	1,893 (73.9%)	491 (70.1%)		374 (76.3%)	
≥41	506 (19.8%)	133 (19.0%)		102 (20.8%)	
Missing	14 (0.5%)	0 (0.0%)		0 (0.0%)	
**Birth weight (g)**			0.07		0.02
<2500	213 (8.3%)	72 (10.3%)		24 (4.9%)	
2500-3499	1,541 (60.1%)	426 (60.9%)		307 (62.7%)	
≥3500	794 (31.0%)	202 (28.9%)		159 (32.4%)	
Missing	14 (0.5%)	0 (0.0%)		0 (0.0%)	
**Sex**			0.02		0.09
Female	1,246 (48.6%)	306 (43.7%)		219 (44.7%)	
Male	1,302 (50.8%)	394 (56.3%)		271 (55.3%)	
Missing	14 (0.5%)	0 (0.0%)		0 (0.0%)	
**Maternal ethnic group**			0.02		<0.01
White British	952 (37.2%)	232 (33.1%)		150 (30.6%)	
Pakistani origins	1,251 (48.8%)	384 (54.9%)		282 (57.6%)	
Other	357 (13.9%)	84 (12.0%)		58 (11.8%)	
Missing	2 (0.1%)	0 (0.0%)		0 (0.0%)	
**Parity**			0.22		0.04
0	920 (35.9%)	227 (32.4%)		145 (29.6%)	
1	732 (28.6%)	203 (29.0%)		147 (30.0%)	
2	441 (17.2%)	124 (17.7%)		95 (19.4%)	
≥3	403 (15.7%)	129 (18.4%)		92 (18.8%)	
Missing	66 (2.6%)	4 (0.6%)		2 (0.4%)	
**Gestational diabetes**			0.15		0.32
No	2,312 (90.2%)	623 (89.0%)		438 (89.4%)	
Yes	234 (9.1%)	77 (11.0%)		52 (10.6%)	
Missing	16 (0.6%)	0 (0.0%)		0 (0.0%)	
**Hypertension****			0.23		0.23
No	2,244 (87.6%)	629 (89.9%)		446 (91.0%)	
Yes	177 (6.9%)	40 (5.7%)		19 (3.9%)	
Missing	141 (5.5%)	0 (0.0%)		0 (0.0%)	
**Time of birth**					
Oct-Dec	657 (25.6%)	193 (27.6%)	0.57	143 (29.2%)	0.13
Jan-Mar	662 (25.8%)	168 (24.0%)		123 (25.1%)	
Apr-Jun	617 (24.1%)	161 (23.0%)		97 (19.8%)	
Jul-Sep	626 (24.4%)	178 (25.4%)		127 (25.9%)	

ALL-IN = Allergy and Infection study. Columns 2, 3 and 5 present number and % of children in ALL-IN study, sampled children with cord bloods and with all 3 blood measurements, respectively, tabulated by each risk factor category. Columns 3 and 5 show p-values for Chi squared test comparing children with blood samples with ALL-IN participants, excluding missing data category. *We oversampled children born prematurely **Hypertension included any mention of history of hypertension/pregnancy induced hypertension/ preeclampsia

Supplementary Table S4 – Comparison of questionnaire responses at age 1 and 2 years old in the study sub-cohort with blood samples measured at age 1 and 2, and all participants in the ALL-IN study

	**Aged 1 year old**			**Aged 2 years old**		
**Risk factor**	**ALL-IN children n = 2,562***	**Children with longitudinal blood measurements n=490**	**p-value**	**ALL-IN children n = 2,067***	**Children with longitudinal blood measurements n=490**	**p-value**
**Age at measurement (in months)**						
Mean (95% CI)	13.06 (13.01, 13.10)	13.07 (12.98, 13.17)	0.78	25.60 (25.54, 25.66)	25.60 (25.48, 25.72)	0.92
Missing	11 (0.4%)	2 (0.4%)		0 (0.0%)	0 (0.0%)	
**Time of measurement**						
Oct-Dec	629 (24.6%)	133 (27.1%)	0.60	475 (23.0%)	129 (26.3%)	0.29
Jan-Mar	593 (23.1%)	110 (22.4%)		394 (19.1%)	98 (20.0%)	
Apr-Jun	675 (26.3%)	119 (24.3%)		582 (28.2%)	134 (27.3%)	
Jul-Sep	654 (25.5%)	126 (25.7%)		616 (29.8%)	129 (26.3%)	
Missing	11 (0.4%)	2 (0.4%)		0 (0.0%)	0 (0.0%)	
**Household size**						
2-4 people	1,330 (51.9%)	224 (45.7%)	0.01	1,310 (63.4%)	221 (45.1%)	0.01
5+ people	1,220 (47.6%)	264 (53.9%)		1,243 (60.1%)	268 (54.7%)	
Missing	12 (0.5%)	2 (0.4%)		9 (0.4%)	1 (0.2%)	
**Share a bedroom**						
No	1,897 (74.0%)	386 (78.8%)	0.03	1,470 (71.1%)	386 (78.8%)	<0.01
Yes	652 (25.4%)	102 (20.8%)		597 (28.9%)	104 (21.2%)	
Missing	13 (0.5%)	2 (0.4%)		0 (0.0%)	0 (0.0%)	
**Any formal childcare**						
No	2,104 (82.1%)	407 (83.1%)	0.63	1,566 (75.8%)	381 (77.8%)	0.28
Yes	446 (17.4%)	81 (16.5%)		434 (21.0%)	92 (18.8%)	
Missing	12 (0.5%)	2 (0.4%)		67 (3.2%)	17 (3.5%)	
**Mother-baby activity**						
	**Aged 0-6 months**			**Aged 12-24 moths**		
Rarely	1,795 (70.1%)	363 (74.1%)	0.08	1,124 (54.4%)	277 (56.5%)	0.40
More than once a week	753 (29.4%)	125 (25.5%)		937 (45.3%)	212 (43.3%)	
Missing	14 (0.5%)	2 (0.4%)		6 (0.3%)	1 (0.2%)	
	**Aged 6-12 months**					
Rarely	1,744 (68.1%)	350 (71.4%)	0.15			
More than once a week	806 (31.5%)	138 (28.2%)				
Missing	12 (0.5%)	2 (0.4%)				
**RTI-related contact with healthcare during Nov-Jan before measurement**						
No	1,827 (71.3%)	343 (70.0%)	0.56	1,865 (72.8%)	359 (73.3%)	0.83
Yes	735 (28.7%)	147 (30.0%)		697 (27.2%)	131 (26.7%)	

ALL-IN = Allergy and Infection study, CI=confidence interval. RTI=respiratory tract infections. Columns 2, 3, 5 and 6 present number and % of children according to their categorical responses to questionnaires and means with 95% CIs for continuous responses for children in ALL-IN study and among sampled children with blood measurements at age 1 and 2 years old, respectively. Columns 4 and 7 show p-values for Chi squared test for categorical variables and t-test for continuous variables, comparing distribution of risk factors in sampled children vs ALL-IN participants, excluding missing data category. *Detailed questionnaire data about the family and home environment was collected from 2,562 children at age 1 and 2067 children at age 2 years.

2.2 Descriptive analyses

Supplementary Table S5 – Distribution of mean maternally derived log RSV IgG post-F antibody levels by risk factor category.

**Risk factor category**	**Mean maternally derived log RSV IgG post-F antibody (95% CI)**	**p-value**
**Gestational age (weeks)**		<0.01
<35	5.37 (5.13, 5.60)	
35-36	5.66 (5.37, 5.95)	
37-40	5.84 (5.77, 5.91)	
≥41	5.99 (5.85, 6.13)	
**Birth weight (g)**		0.04
<2500	5.63 (5.43, 5.82)	
2500-3499	5.83 (5.75, 5.91)	
≥3500	5.92 (5.81, 6.04)	
**Sex**		0.59
Female	5.86 (5.76, 5.95)	
Male	5.82 (5.74, 5.90)	
**Maternal ethnic group**		0.39
White British	5.82 (5.71, 5.92)	
Pakistani origins	5.82 (5.73, 5.91)	
Other	5.95 (5.80, 6.10)	
**Parity**		0.04
0	5.73 (5.61, 5.84)	
1	5.94 (5.83, 6.05)	
2	5.91 (5.76, 6.06)	
≥3	5.82 (5.67, 5.97)	
Missing	5.68 (5.33, 6.03)	
**Gestational diabetes**		0.40
No	5.83 (5.76, 5.89)	
Yes	5.91 (5.73, 6.09)	
**Hypertension***		0.40
No	5.83 (5.77, 5.90)	
Yes	5.95 (5.73, 6.17)	
Missing	5.77 (5.53, 6.01)	
**Time of birth**		0.01
Oct-Dec	5.67 (5.55, 5.79)	
Jan-Mar	5.87 (5.75, 6.00)	
Apr-Jun	5.90 (5.78, 6.02)	
Jul-Sep	5.93 (5.80, 6.05)	

CI=confidence interval, IgG post-F=immunoglobulin G antibody against RSV post-fusion protein F, log = natural logarithm (base e), RSV=respiratory syncytial virus. Column 2 presents mean maternal log RSV post-F antibody levels. Column 3 presents p-values for one-way analysis of variance (ANOVA) comparing mean maternal log RSV IgG post-F by risk factor categories, excluding missing data category. *Hypertension included any mention of history of hypertension/pregnancy induced hypertension/ preeclampsia

Supplementary Table S6 – Distribution of risk factors in children with primary RSV infection vs never infected at age 1 and 2 years old

	Aged 1 year old			Aged 2 years old		
**Risk factor**	**Never RSV infected at age 1 year old**	**First RSV infected at age 1 year old**	**p-value**	**Never RSV infected at age 2 years old**	**First RSV infected at age 2 years old**	**p-value**
**Age at measurement (in months)**						
Mean (95% CI)	12.9	13.2	0.01	25.4	25.5	0.43
	(12.8, 13.1)	(13.1, 13.3)		(25.1, 25.7)	(25.3, 25.8)	
Missing	0	2 (0.8%)		0	0	
**Sex**						
Female	102 (44.0%)	117 (45.3%)	0.76	28 (41.2%)	74 (45.1%)	0.58
Male	130 (56.0%)	141 (54.7%)		40 (58.8%)	90 (54.9%)	
**Ethnicity**						
White British	78 (33.6%)	72 (27.9%)	0.39	20 (29.4%)	58 (35.4%)	0.42
Pakistani	128 (55.2%)	154 (59.7%)		42 (61.8%)	86 (52.4%)	
Other	26 (11.2%)	32 (12.4%)		6 (8.8%)	20 (12.2%)	
**Time of birth**						
Oct-Dec	61 (26.3%)	82 (31.8%)	0.00	12 (17.6%)	49 (29.9%)	0.01
Jan-Mar	52 (22.4%)	71 (27.5%)		12 (17.6%)	40 (24.4%)	
Apr-Jun	37 (15.9%)	60 (23.3%)		18 (26.5%)	19 (11.6%)	
Jul-Sep	82 (35.3%)	45 (17.4%)		26 (38.2%)	56 (34.1%)	
**Parity**						
0	85 (36.6%)	60 (23.3%)	0.01	33 (48.5%)	52 (31.7%)	0.06
1	58 (25.0%)	89 (34.5%)		11 (16.2%)	47 (28.7%)	
2	47 (20.3%)	48 (18.6%)		14 (20.6%)	33 (20.1%)	
≥3	39 (16.8%)	53 (20.5%)		9 (13.2%)	30 (18.3%)	
missing	3 (1.3%)	8 (3.1%)		1 (1.5%)	2 (1.2%)	
**Household size**						
2-4 people	108 (46.6%)	116 (45.0%)	0.78	33 (48.5%)	74 (45.1%)	0.64
5+ people	124 (53.4%)	140 (54.3%)		35 (51.5%)	90 (54.9%)	
Missing	0 (0.0%)	2 (0.8%)		0 (0.0%)	0 (0.0%)	
**Share a bedroom**						
No	186 (80.2%)	200 (77.5%)	0.58	51 (75.0%)	130 (79.3%)	0.47
Yes	46 (19.8%)	56 (21.7%)		17 (25.0%)	34 (20.7%)	
Missing	0 (0.0%)	2 (0.8%)		0 (0.0%)	0 (0.0%)	
**Any formal childcare**						
No	201 (86.6%)	206 (79.8%)	0.07	56 (82.4%)	127 (77.4%)	0.18
Yes	31 (13.4%)	50 (19.4%)		8 (11.8%)	32 (19.5%)	
Missing	0 (0.0%)	2 (0.8%)		4 (5.9%)	5 (3.0%)	
**Mother-baby activity**						
	**Aged 0-6 months**			**Aged 12-24 moths**		
Rarely	180 (77.6%)	183 (70.9%)	0.12	41 (60.3%)	98 (59.8%)	0.98
More than once a week	52 (22.4%)	73 (28.3%)		27 (39.7%)	65 (39.6%)	
Missing	0 (0.0%)	2 (0.8%)		0 (0.0%)	1 (0.6%)	
	**Aged 6-12 months**					
Rarely	170 (73.3%)	180 (69.8%)	0.47			
More than once a week	62 (26.7%)	76 (29.5%)				
Missing	0 (0.0%)	2 (0.8%)				

CI=confidence interval, RSV=respiratory syncytial virus. Columns 2, 3, 5 and 6 present the distribution of risk factors according to infection status at age 1 and 2 years old (number and % of children according to categorical risk factors and mean and 95% CI for continuous variables). Columns 4 and 7 show p-values for Chi squared test for categorical variables and t-test for continuous variables, comparing distribution of risk factors by infection status at ages 1 and 2 years old, excluding missing data category.

2.3 Sensitivity analyses

Supplementary Table S7 – Risk ratios for primary RSV infection at age 1 year old according to risk factors of interest – comparison of main results and 3 sensitivity analyses

**Risk factor**	**Main analyses**	**Sensitivity analysis 1:** indicate children who had RTI-related contact with healthcare in Oct-Feb aged <6 months to be infected in first year of life	**Sensitivity analysis 2:** indicate children who had RTI-related contact with healthcare in Nov-Jan aged <6 months to be infected in first year of life	**Sensitivity analysis 3:** pool together results analyses of 50 simulated infection statuses to allow for misclassification error
All included children	477	477	477	477
Number of infected children	248	288	277	249 (SD=2.0)
% infected (95% CI)	52% (47%-57%)	60% (56%-65%)	58% (53%-63%)	
**Sex**				
Male (baseline)	1	1	1	1
Female	1.07 (0.91, 1.27)	1.04 (0.90, 1.20)	1.07 (0.92, 1.25)	1.07 (0.90, 1.27)
**Ethnic group**				
White British (baseline)	1	1	1	1
Pakistani origins	1.23 (0.98, 1.55)	1.17 (0.97, 1.42)	1.27 (1.03, 1.56)	1.21 (0.96, 1.53)
Other	1.27 (0.95, 1.69)	1.15 (0.88, 1.49)	1.26 (0.96, 1.64)	1.24 (0.92, 1.66)
**Annual quarter of birth**				
Jul–Sep (baseline)	1	1	1	1
Oct–Dec	1.57 (1.19, 2.08)	1.14 (0.93, 1.40)	1.16 (0.94, 1.45)	1.53 (1.15, 2.03)
Jan–Mar	1.57 (1.18, 2.09)	1.05 (0.84, 1.31)	1.10 (0.88, 1.39)	1.56 (1.17, 2.08)
Apr–Jun	1.63 (1.22, 2.18)	1.23 (1.00, 1.52)	1.25 (1.00, 1.56)	1.58 (1.17, 2.12)
**Age at measurement** (centred around 12 months)	1.08 (1.00, 1.16)	1.04 (0.98, 1.11)	1.04 (0.98, 1.12)	1.08 (1.00, 1.16)
**Parity**				
0 (baseline)	1	1	1	1
1	1.49 (1.18, 1.87)	1.46 (1.20, 1.79)	1.48 (1.20, 1.82)	1.48 (1.17, 1.86)
2	1.22 (0.93, 1.60)	1.17 (0.92, 1.50)	1.22 (0.96, 1.57)	1.22 (0.93, 1.61)
3+	1.38 (1.06, 1.79)	1.43 (1.14, 1.79)	1.41 (1.11, 1.78)	1.40 (1.07, 1.82)
**Attending any formal care**				
No (baseline)	1	1	1	1
Yes	1.36 (1.09, 1.69)	1.19 (0.98, 1.45)	1.30 (1.06, 1.60)	1.33 (1.06, 1.66)

CI = confidence interval, RTI = respiratory tract infection, RSV = respiratory syncytial virus, SD = standard deviation. All columns present adjusted risk ratios for primary RSV infection by each risk factor at age <1, estimated from Poisson regression models with robust error variances calculated using sandwich estimator.

Supplementary Table S8 – Risk ratios for primary RSV infection at age 2 years old according to risk factors of interest – comparison of main results and 3 sensitivity analyses

**Risk factor**	**Main analyses**	**Sensitivity analysis 1:** indicate children who had RTI-related contact with healthcare in Oct-Feb aged <6 months to be infected in first year of life (and exclude them from analyses at age 1-2 years)	**Sensitivity analysis 2:** indicate children who had RTI-related contact with healthcare in Nov-Jan aged <6 months to be infected in first year of life (and exclude them from analyses at age 1-2 years)	**Sensitivity analysis 3:** pool together results analyses of 50 simulated infection statuses to allow for misclassification error
All included children	229	189	200	228
Number of infected children	162	136	143	162 (SD=2.4)
% newly infected of all children in the cohort (95% CI)	34% (30%-38%)	29% (24%-33%)	30% (26%-34%)	
**Sex**				
Male (baseline)	1	1	1	1
Female	1.12 (0.95, 1.32)	1.14 (0.96, 1.37)	1.13 (0.94, 1.35)	1.10 (0.92, 1.30)
**Ethnic group**				
White British (baseline)	1	1	1	1
Pakistani origins	0.88 (0.73, 1.05)	0.84 (0.69, 1.03)	0.87 (0.71, 1.06)	0.86 (0.72, 1.04)
Other	1.01 (0.80, 1.28)	1.00 (0.78, 1.28)	1.02 (0.80, 1.31)	1.02 (0.80, 1.29)
**Annual quarter of birth**				
Jul–Sep (baseline)	1	1	1	1
Oct–Dec	1.23 (1.02, 1.48)	1.25 (0.99, 1.57)	1.26 (1.01, 1.55)	1.23 (0.97, 1.56)
Jan–Mar	1.12 (0.89, 1.41)	1.16 (0.91, 1.49)	1.14 (0.89, 1.45)	1.12 (0.93, 1.35)
Apr–Jun	0.77 (0.54, 1.09)	0.94 (0.66, 1.34)	0.88 (0.62, 1.25)	0.81 (0.64, 1.03)
**Age at measurement** (centred around 24 months)	1.02 (0.96, 1.08)	1.04 (0.98, 1.11)	1.03 (0.97, 1.09)	1.03 (0.97, 1.09)
**Parity**				
0 (baseline)	1	1	1	1
1	1.49 (1.18, 1.87)	1.46 (1.20, 1.79)	1.48 (1.20, 1.82)	1.48 (1.17, 1.86)
2	1.22 (0.93, 1.60)	1.17 (0.92, 1.50)	1.22 (0.96, 1.57)	1.22 (0.93, 1.61)
3+	1.38 (1.06, 1.79)	1.43 (1.14, 1.79)	1.41 (1.11, 1.78)	1.40 (1.07, 1.82)

CI = confidence interval, RTI –respiratory tract infection, RSV = respiratory syncytial virus, SD = standard deviation. All columns present risk ratios for primary RSV infection by each risk factor at age 1 and 2 years old, respectively, estimated from Poisson regression models with robust error variances calculated using sandwich estimator.

2.4 Secondary analyses using RSV Immunoglobulin G (IgG) antibodies against proteins Ga and Gb

2.4.1 Maternal RSV antibody concentrations

Supplementary Table S9 – Risk factors associated with increase in mean maternal RSV IgG Ga and Gb antibody levels

Risk factor category	Adjusted ratios of mean maternal RSV IgG Ga (95% CI)	Adjusted ratios of mean maternal RSV IgG Gb (95% CI)
**Gestational age (weeks)**		
<35	0.57 (0.41, 0.80)	0.56 (0.40, 0.78)
35-36	1.03 (0.79, 1.35)	0.79 (0.60, 1.05)
37-40 (baseline)	1	1
≥41	1.00 (0.84, 1.18)	1.04 (0.87, 1.24)
**Parity**		
0 (baseline)	1	1
1	1.34 (1.13, 1.58)	1.42 (1.20, 1.69)
2	1.35 (1.11, 1.65)	1.36 (1.11, 1.66)
≥3	1.51 (1.24, 1.84)	1.45 (1.18, 1.77)
**Ethnicity**		
White British (baseline)	1	1
Pakistani origins	1.19 (1.03, 1.39)	1.34 (1.15, 1.56)
Other	1.19 (0.95, 1.49)	1.43 (1.13, 1.80)
**Time of birth**	1	1
Jan-Mar	1.30 (1.08, 1.57)	1.24 (1.02, 1.50)
Apr-Jun	1.33 (1.11, 1.61)	1.46 (1.21, 1.77)
Jul-Sep	1.22 (1.01, 1.46)	1.38 (1.14, 1.66)
Oct-Dec (baseline)	1	1
**Mean maternal RSV IgG levels at baseline (in arbitrary units/mL)**	6.94 (5.73, 8.40)	7.91 (6.50, 9.63)

CI=confidence interval, IgG Ga and Gb = immunoglobulin G (IgG) antibody against attachment protein G for RSV strands A and B, RSV=respiratory syncytial virus. Columns 2 and 3 present exponentiated results from the log-linear model, reflecting proportional change in maternal RSV IgG Ga and GB levels, respectively, for each risk factor category vs the baseline.

2.4.2 RSV antibody concentrations at ages 1 and 2 years old

The distribution of IgG Ga and Gb at age 1 did not show a clear bimodal pattern unlike IgG post-F (supplementary figure 2), indicating an overlap in distributions of antibody concentrations in RSV-infected and never infected children. This led to less extreme values of probabilities of infection and more uncertainty around the derived infection status (supplementary figure 3).

Definition of "RSV infection" using each of the 3 antibody types agreed for 58% of children, and further 33% showed agreement between IgG post-F and either Ga or Gb antibodies (supplementary table 10). Since IgG Ga and Gb antibodies are less immunogenic than IgG post-F, resulting in lower sensitivity and specificity,[4] we did not re-calculate risk ratios based on infection indicator from these models. At age 2 years, the distribution of IgG Ga and Gb proteins was approximately normal (supplementary figure 2), therefore we did not fit FMM to samples at that age.

Supplementary Figure S2 – Distribution of log RSV IgG Ga and Gb at birth, age 1 and 2 years old

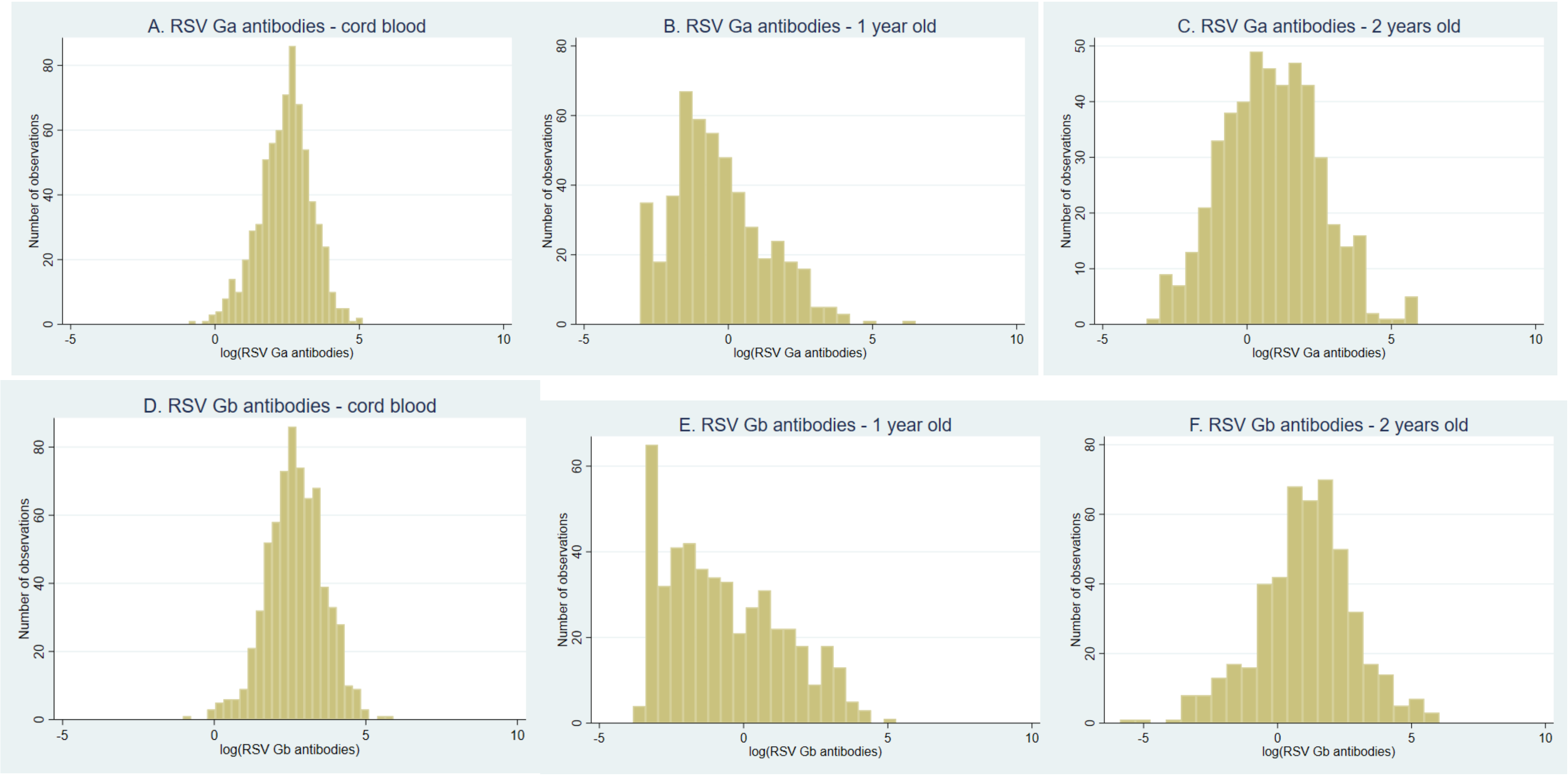

IgG Ga and Gb = immunoglobulin G (IgG) antibody against attachment protein G for RSV strands A and B, RSV=respiratory syncytial virus. Note that figures A and D are based on 700 blood samples, and figures B, C, E and F are based on 490 blood samples. IgG Ga and Gb concentrations were quantified in arbitrary units/ml.

Supplementary Figure S3 – Posterior probabilities of RSV infection at age 1 and 2 years old according to IgG antibodies Ga and Gb

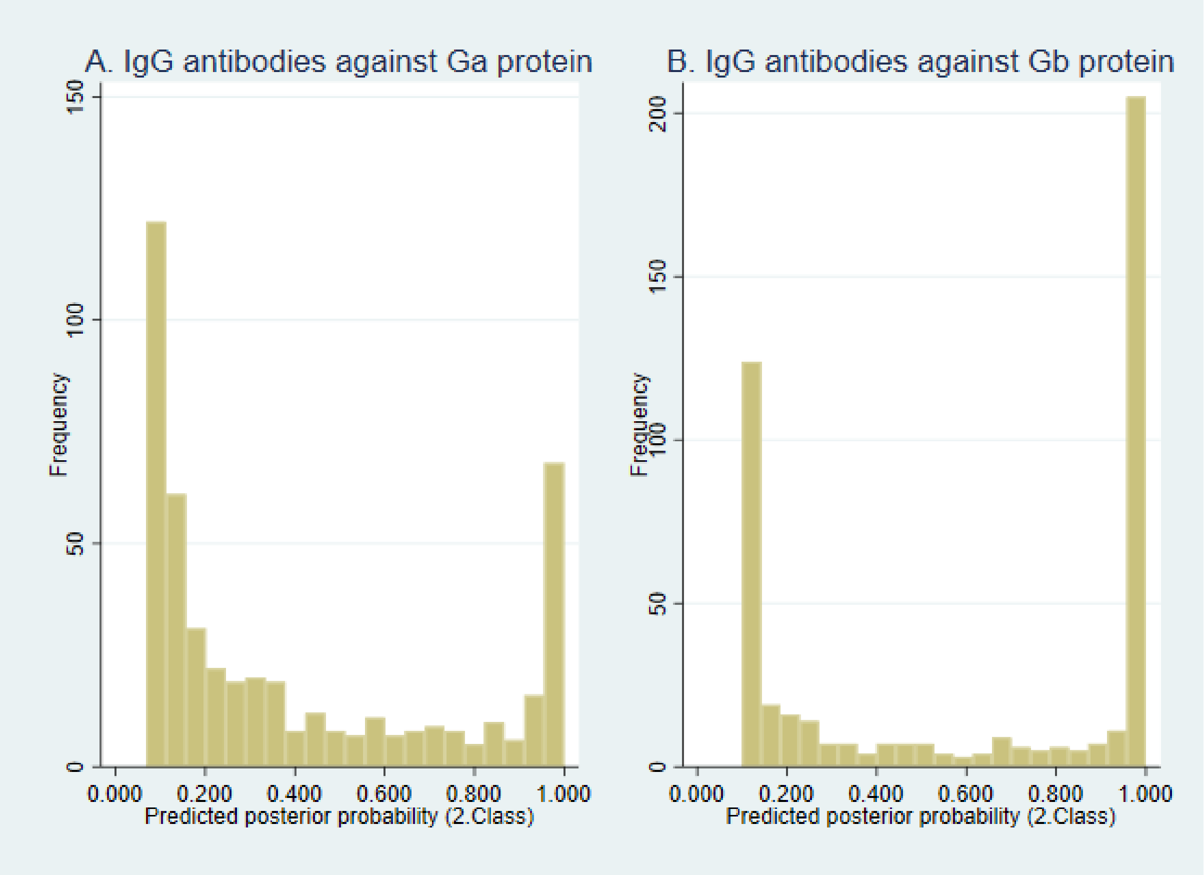

IgG Ga and Gb = immunoglobulin G (IgG) antibody against attachment protein G for RSV strands A and B, RSV=respiratory syncytial virus.

Supplementary Table S10 – Number and % of children indicated as infected according to IgG post-F, Ga and Gb.

Presence of evidence of primary RSV infection aged <1 year according to antibody type:			Number of children n=490	% of all children
**IgG post-F**	**IgG Ga**	**IgG Gb**		
Yes	Yes	Yes	118	24%
Yes	Yes	No	15	3%
Yes	No	Yes	99	20%
Yes	No	No	26	5%
No	Yes	Yes	24	5%
No	Yes	No	9	2%
No	No	Yes	33	7%
No	No	No	166	34%

IgG post-F=immunoglobulin G antibody against RSV post-fusion protein F, IgG Ga and Gb = immunoglobulin G antibody against attachment protein G for RSV strands A and B, RSV=respiratory syncytial virus.

References

1. Pembrey L, Waiblinger D, Griffiths P, Patel M, Azad R, Wright J. Cytomegalovirus, Epstein-Barr virus and varicella zoster virus infection in the first two years of life: a cohort study in Bradford, UK. *BMC Infect Dis* [Internet]. BioMed Central; 2017 Dec 21 [cited 2019 Feb 8];**17**(1):220. Available from: http://bmcinfectdis.biomedcentral.com/articles/10.1186/s12879-017-2319-7

2. Ohuma EO, Okiro EA, Ochola R, et al. The natural history of respiratory syncytial virus in a birth cohort: The influence of age and previous infection on reinfection and disease. *Am J Epidemiol*. 2012;**176**(9):794–802.

3. Ochola R, Sande C, Fegan G, et al. The level and duration of RSV-specific maternal IgG in infants in kilifi Kenya. Ng LFP, editor. *PLoS One* [Internet]. Public Library of Science; 2009 Dec 2 [cited 2018 Oct 15];**4**(12):4–9. Available from: http://dx.plos.org/10.1371/journal.pone.0008088

4. Schepp RM, Haan CAM de, Wilkins D, et al. Development and Standardization of a High-Throughput Multiplex Immunoassay for the Simultaneous Quantification of Specific Antibodies to Five Respiratory Syncytial Virus Proteins. Pasetti MF, editor. *mSphere*. American Society for Microbiology Journals; 2019 Apr 24;**4**(2):e00236-19.

5. Wright J, Small N, Raynor P, et al. Cohort profile: The born in bradford multi-ethnic family cohort study. *Int J Epidemiol*. 2013;

6. Born in Bradford. THE BORN IN BRADFORD PLATFORM [Internet]. [cited 2019 Apr 10]. Available from: https://borninbradford.nhs.uk/wp-content/uploads/BiB_Data_Summary_slides.pdf

7. World Health Organization. International statistical classification of diseases and related health problems, 10th revision. Volume 2: instruction manual. 2010th ed. Geneva: WHO Library Cataloguing-in-Publication Data International; 2011.

8. The Phoenix Partnership (TPP). TPP | SystmOne [Internet]. [cited 2019 Mar 25]. Available from: https://www.tpp-uk.com/products/systmone

9. Bishop C, Small N, Mason D, et al. Improving case ascertainment of congenital anomalies: findings from a prospective birth cohort with detailed primary care record linkage. *BMJ Paediatr Open* [Internet]. 2017 Nov 12;**1**(1):e000171. Available from: http://bmjpaedsopen.bmj.com/lookup/doi/10.1136/bmjpo-2017-000171

10. Joint Formulary Committee. British national formulary : book 74. London: London : BMJ Group and Pharmaceutical Press; 2017.

11. Petersen I, Hayward AC. Antibacterial prescribing in primary care. *J Antimicrob Chemother* [Internet]. Narnia; 2007 Aug 1 [cited 2019 Mar 25];**60**(suppl_1):i43–i47. Available from: http://academic.oup.com/jac/article/60/suppl_1/i43/999396/Antibacterial-prescribing-in-primary-care

12. Hardelid P, Ghebremichael-Weldeselassie Y, Whitaker H, Rait G, Gilbert R, Petersen I. Effectiveness of live attenuated influenza vaccine in preventing amoxicillin prescribing in preschool children: a self-controlled case series study. *J Antimicrob Chemother*. 2018 Mar;**73**(3):779–786.
